# Supplementary material for: Epigenetic Regulation in Exposome-Induced Tumorigenesis: Emerging Roles of ncRNAs
Source: Biomolecules. 2022 Mar 28;12(4):513. doi: 10.3390/biom12040513 (PMC9032613; doi:10.3390/biom12040513)
Supplement: Supplementary file 1 [file biomolecules-12-00513-s001.zip › Olmedo-Suarez et al_Supplementary Material_Table References.pdf]

References:

Agency for Toxic Substances and Disease Registry. (2020). ToxFAQsTM for DDT, DDE, and DDD. https://wwwn.cdc.gov/TSP/ToxFAQs/ToxFAQsDetails.aspx?faqid=80&toxid=20

Agency for Toxic Substances and Disease Registry. (2014). *Polychlorinated Biphenyls (PCBs) Toxicity*. https://www.atsdr.cdc.gov/csem/polychlorinated-biphenyls/adverse_health.html

American Cancer Society. (2022). *Perfluorooctanoic Acid (PFOA), Teflon, and Related Chemicals*. https://www.cancer.org/cancer/cancer-causes/teflon-and-perfluorooctanoic-acid-pfoa.html

Alli, L.(2015).Blood level of cadmium and lead in occupationally exposed persons in Gwagwalada, Abuja, Nigeria. *Interdisciplinary Toxicology*,8(3) 146-150. https://doi.org/10.1515/intox-2015-0022

Alvarado, A; Blanco, R. & Mora, E. (2002). El cromo como elemento esencial en los humanos. *Revista Costarricense de Ciencias Médicas,* 23(1-2), 55-68.

Anyanwu, B. O., & Orisakwe, O. E. (2020). Current mechanistic perspectives on male reproductive toxicity induced by heavy metals. Journal of environmental science and health. *Part C, Toxicology and Carcinogenesis*, 38(3), 204–244. https://doi.org/10.1080/26896583.2020.1782116

Avissar-Whiting, M., Veiga, K. R., Uhl, K. M., Maccani, M. A., Gagne, L. A., Moen, E. L., & Marsit, C. J. (2010). Bisphenol A exposure leads to specific microRNA alterations in placental cells. *Reproductive Toxicology* (Elmsford, N.Y.), 29(4), 401–406. https://doi.org/10.1016/j.reprotox.2010.04.004

Awadalla, A., Mortada, W. I., Abol-Enein, H., & Shokeir, A. A. (2020). Correlation between blood levels of cadmium and lead and the expression of microRNA-21 in Egyptian bladder cancer patients. *Heliyon*, 6(12), e05642. https://doi.org/10.1016/j.heliyon.2020.e05642

Bae, H. S., Ryu, D. Y., Choi, B. S., & Park, J. D. (2013). Urinary Arsenic Concentrations and their Associated Factors in Korean Adults. *Toxicological Research*, 29(2), 137–142. https://doi.org/10.5487/TR.2013.29.2.137

Baker, B. A., Cassano, V. A., Murray, C., & ACOEM Task Force on Arsenic Exposure (2018). Arsenic Exposure, Assessment, Toxicity, Diagnosis, and Management: Guidance for Occupational and Environmental Physicians. *Journal of Occupational and Environmental Medicine*, 60(12), e634–e639. https://doi.org/10.1097/JOM.0000000000001485

Beck, R., Bommarito, P., Douillet, C., Kanke, M., Del Razo, L. M., García-Vargas, G., Fry, R. C., Sethupathy, P., & Stýblo, M. (2018). Circulating miRNAs Associated with Arsenic Exposure. *Environmental Science & Technology*, 52(24), 14487–14495. https://doi.org/10.1021/acs.est.8b06457

Blockhuys, S., & Wittung-Stafshede, P.. (2017). Roles of Copper-Binding Proteins in Breast Cancer. *International Journal of Molecular Sciences,* 18(4), 871. https://doi.org/10.3390/ijms18040871

Boffetta, P., Jourenkova, N. & Gustavsson, P. (1997). Riesgo de cáncer por exposición ocupacional y ambiental a hidrocarburos aromáticos policíclicos. *Cancer Causes Control* 8, 444–472

Born, S.C. (2012). Chapter 62. dioxins. Olson K.R.(Ed.), Poisoning & Drug Overdose, 6e. McGraw Hill. https://accessmedicine.mhmedical.com/content.aspx?bookid=391&sectionid=42069876

Bouwman, H., Becker, P. J., & Schutte, C. H. (1994). Malaria control and longitudinal changes in levels of DDT and its metabolites in human serum from KwaZulu. *Bulletin of the World Health Organization*, 72(6), 921–930.

Brucker, N., Charão, M. F., Moro, A. M., Ferrari, P., Bubols, G., Sauer, E., Fracasso, R., Durgante, J., Thiesen, F. V., Duarte, M. M., Gioda, A., Castro, I., Saldiva, P. H., & Garcia, S. C. (2014). Atherosclerotic process in taxi drivers occupationally exposed to air pollution and co-morbidities. *Environmental Research*, 131, 31–38. https://doi.org/10.1016/j.envres.2014.02.012

Burstyn, I., Kromhout, H., Partanen, T., Svane, O., Langård, S., Ahrens, W., Kauppinen, T., Stücker, I., Shaham, J., Heederik, D., Ferro, G., Heikkilä, P., Hooiveld, M., Johansen, C., Randem, B. G., & Boffetta, P. (2005). Polycyclic aromatic hydrocarbons and fatal ischemic heart disease. *Epidemiology* (Cambridge, Mass.), 16(6), 744–750. https://doi.org/10.1097/01.ede.0000181310.65043.2f

CDC. (2009). *Polycyclic Aromatic Hydrocarbons (PAHs)*. Centers for Disease Control and Prevention. https://www.epa.gov/sites/default/files/2014-03/documents/pahs_factsheet_cdc_2013.pdf

CDC. (2009a). *Dichlorodiphenyltrichloroethane (DDT)*. Centers for Disease Control and Prevention.

https://www.cdc.gov/biomonitoring/pdf/ddt_factsheet.pdf

CDC. (2014). *Lead compounds (as Pb)*. Centers for Disease Control and Prevention.https://www.cdc.gov/niosh/idlh/7439921.html

CDC. (2014a). *Smoking and cardiovascular disease*. Centers for Disease Control and Prevention. https://www.cdc.gov/tobacco/data_statistics/sgr/50th-anniversary/pdfs/fs_smoking_CVD_508.pdf

CDC. (2016). *ToxFAQs™ - DDT, DDE y DDD*. Centers for Disease Control and Prevention. https://www.atsdr.cdc.gov/es/toxfaqs/es_tfacts35.html

CDC. (2017). *Bisphenol A (BPA) Factsheet*. Centers for Disease Control and Prevention. https://www.cdc.gov/biomonitoring/BisphenolA_FactSheet.html

CDC. (2020). *Sources of Lead Exposure*. Centers for Disease Control and Prevention. https://www.cdc.gov/nceh/lead/prevention/sources.htm

CDC. (2021). *Health Effects of Cigarette Smoking*. Centers for Disease Control and Prevention. https://www.cdc.gov/tobacco/data_statistics/fact_sheets/health_effects/effects_cig_smoking/index.htm

CDC. (2021a). *Phthalates Factsheet*. Centers for Disease Control and Prevention. https://www.cdc.gov/biomonitoring/Phthalates_FactSheet.html

Chuang, S. C., Chen, H. C., Sun, C. W., Chen, Y. A., Wang, Y. H., Chiang, C. J., Chen, C. C., Wang, S. L., Chen, C. J., & Hsiung, C. A. (2020). Phthalate exposure and prostate cancer in a population-based nested case-control study. *Environmental Research*, 181, 108902. https://doi.org/10.1016/j.envres.2019.108902

Cobellis, L., Colacurci, N., Trabucco, E., Carpentiero, C., & Grumetto, L. (2009). Measurement of bisphenol A and bisphenol B levels in human blood sera from healthy and endometriotic women. Biomedical chromatography : *BMC*, 23(11), 1186–1190. https://doi.org/10.1002/bmc.1241

Cohn, B. A., Wolff, M. S., Cirillo, P. M., & Sholtz, R. I. (2007). DDT and breast cancer in young women: new data on the significance of age at exposure. *Environmental Health Perspectives*, 115(10), 1406–1414. https://doi.org/10.1289/ehp.10260

Collaborative on Health and the Environment. (2019). *Arsenic.* https://www.healthandenvironment.org/environmental-health/environmental-risks/chemical-environment-overview/arsenic

Craig, S. (2012). Chapter 78. Glyphosate. In: Olson, K.R. *Poisoning & Drug Overdose*, 6e. McGrawHill.

Dann, A. B., & Hontela, A. (2011). Triclosan: environmental exposure, toxicity and mechanisms of action. *Journal of applied Toxicology : JAT*, 31(4), 285–311. https://doi.org/10.1002/jat.1660

Deng, Q., Huang, S., Zhang, X., Zhang, W., Feng, J., Wang, T., Hu, D., Guan, L., Li, J., Dai, X., Deng, H., Zhang, X., & Wu, T. (2014). Plasma microRNA expression and micronuclei frequency in workers exposed to polycyclic aromatic hydrocarbons. *Environmental Health Perspectives*, 122(7), 719–725. https://doi.org/10.1289/ehp.1307080

Dinwiddie, M., Terry, P., & Chen, J.. (2014). Recent Evidence Regarding Triclosan and Cancer Risk. International *Journal of Environmental Research and Public Health*, 11(2), 2209–2217. https://doi.org/10.3390/ijerph110202209

Dioni, L., Sucato, S., Motta, V., Iodice, S., Angelici, L., Favero, C., Cavalleri, T., Vigna, L., Albetti, B., Fustinoni, S., Bertazzi, P., Pesatori, A., & Bollati, V. (2017). Urinary chromium is associated with changes in leukocyte miRNA expression in obese subjects. *European Journal of Clinical Nutrition*, 71(1), 142–148. https://doi.org/10.1038/ejcn.2016.197

Du, Z., Chai, X., Li, X., Ren, G., Yang, X., & Yang, Z. (2022). Nano-CuO causes cell damage through activation of dose-dependent autophagy and mitochondrial lncCyt b-AS/ND5-AS/ND6-AS in SH-SY5Y cells. *Toxicology Mechanisms and Methods,* 32(1), 37–48. https://doi.org/10.1080/15376516.2021.1964665

Falagan-Lotsch, P., & Murphy, C. J.. (2020). Network-based analysis implies critical roles of microRNAs in the long-term cellular responses to gold nanoparticles. *Nanoscale*, 12(41), 21172–21187. https://doi.org/10.1039/d0nr04701e

Gao, H., Yang, B. J., Li, N., Feng, L. M., Shi, X. Y., Zhao, W. H., & Liu, S. J. (2015). Bisphenol A and hormone-associated cancers: current progress and perspectives. *Medicine*, 94(1), e211. https://doi.org/10.1097/MD.0000000000000211

Garshick, E., Laden, F., Hart, J. E., Davis, M. E., Eisen, E. A., & Smith, T. J. (2012). Lung cancer and elemental carbon exposure in trucking industry workers. *Environmental Health Perspectives*, 120(9), 1301–1306. https://doi.org/10.1289/ehp.1204989

Gaum, P. M., Gube, M., Schettgen, T., Putschögl, F. M., Kraus, T., Fimm, B., & Lang, J.. (2017). Polychlorinated biphenyls and

depression: cross-sectional and longitudinal investigation of a dopamine-related Neurochemical path in the German HELPcB surveillance program. *Environmental Health*, 16(1). https://doi.org/10.1186/s12940-017-0316-3

Gerona, R. R., Pan, J., Zota, A. R., Schwartz, J. M., Friesen, M., Taylor, J. A., Hunt, P. A., & Woodruff, T. J. (2016). Direct measurement of Bisphenol A (BPA), BPA glucuronide and BPA sulfate in a diverse and low-income population of pregnant women reveals high exposure, with potential implications for previous exposure estimates: a cross-sectional study. *Environmental Health : a global access science source*, 15, 50. https://doi.org/10.1186/s12940-016-0131-2

Gonskikh, Y., Gerstl, M., Kos, M., Borth, N., Schosserer, M., Grillari, J., & Polacek, N.. (2020). Modulation of mammalian translation by a ribosome-associated tRNA half. *RNA Biology*, 17(8), 1125–1136. https://doi.org/10.1080/15476286.2020.1744296

Government of Spain. (2020). DDT. https://prtr-es.es/DDT,15620,11,2007.html

Harari, F., Barregard, L., Östling, G., Sallsten, G., Hedblad, B., Forsgard, N., Borné, Y., Fagerberg, B., & Engström, G. (2019). Blood Lead Levels and Risk of Atherosclerosis in the Carotid Artery: Results from a Swedish Cohort. *Environmental Health Perspectives*, 127(12), 127002. https://doi.org/10.1289/EHP5057

Hollensteiner, J., Schneider, D., Poehlein, A., & Daniel, R.. (2020). Complete Genome of Roseobacter ponti DSM 106830T. *Genome Biology and Evolution*, 12(7), 1013–1018. https://doi.org/10.1093/gbe/evaa114

Hong, Y. S., Song, K. H., & Chung, J. Y. (2014). Health effects of chronic arsenic exposure. *Journal of Preventive Medicine and Public Health = Yebang Uihakhoe chi*, 47(5), 245–252. https://doi.org/10.3961/jpmph.14.035

Iida, T., & Todaka, T. (2003). Measurement of dioxins in human blood: improvement of analytical method. *Industrial health*, 41(3), 197–204. https://doi.org/10.2486/indhealth.41.197

Kalinina, T.S., Kononchuk, V.V., Ovchinnikov, V.Y. et al. (2018). Expression of the miR-190 family is increased under DDT exposure in vivo and in vitro. *Molecular Biology Reports*, 45, 1937–1945 https://doi.org/10.1007/s11033-018-4343-0

Kim, J. H., Cho, Y. H., & Hong, Y. C. (2020). MicroRNA expression in response to bisphenol A is associated with high blood pressure. *Environment International*, 141, 105791. https://doi.org/10.1016/j.envint.2020.105791

Konieczna, A., Rutkowska, A., & Rachoń, D. (2015). Health risk of exposure to Bisphenol A (BPA). *Roczniki Panstwowego Zakladu Higieny*, 66(1), 5–11.

Lee, H. W., Jose, C. C., & Cuddapah, S. (2021). Epithelial-mesenchymal transition: Insights into nickel-induced lung diseases. *Seminars in Cancer Biology*, 76, 99–109. https://doi.org/10.1016/j.semcancer.2021.05.020

Lee, T.-W., Kim, D. H., & Ryu, J. Y.. (2020). Association between urinary polycyclic aromatic hydrocarbons and hypertension in the Korean population: data from the Second Korean National Environmental Health Survey (2012–2014). *Scientific Reports*, 10(1). https://doi.org/10.1038/s41598-020-74353-w

Lim, J. H., Song, M. K., Cho, Y., Kim, W., Han, S. O., & Ryu, J. C. (2017). Comparative analysis of microRNA and mRNA expression profiles in cells and exosomes under toluene exposure. *Toxicology in vitro : an International Journal published in association with BIBRA*, 41, 92–101. https://doi.org/10.1016/j.tiv.2017.02.020

López-Carrillo, L., Hernández-Ramírez, R. U., Calafat, A. M., Torres-Sánchez, L., Galván-Portillo, M., Needham, L. L., Ruiz-Ramos, R., & Cebrián, M. E.. (2010). Exposure to Phthalates and Breast Cancer Risk in Northern Mexico. *Environmental Health Perspectives,* 118(4), 539–544. https://doi.org/10.1289/ehp.0901091

Luz, A., DeLeo, P., Pechacek, N., & Freemantle, M. (2020). Human health hazard assessment of quaternary ammonium compounds: Didecyl dimethyl ammonium chloride and alkyl (C12-C16) dimethyl benzyl ammonium chloride. *Regulatory Toxicology and Pharmacology : R*TP, 116, 104717. https://doi.org/10.1016/j.yrtph.2020.104717

Messerlian, C., Wylie, B. J., Mínguez-Alarcón, L., Williams, P. L., Ford, J. B., Souter, I. C., Calafat, A. M., & Hauser, R.. (2016). Urinary Concentrations of Phthalate Metabolites and Pregnancy Loss Among Women Conceiving with Medically Assisted Reproduction. *Epidemiology*, 27(6), 879–888. https://doi.org/10.1097/ede.0000000000000525

Mohamed, F., Gawarammana, I., Robertson, T. A., Roberts, M. S., Palangasinghe, C., Zawahir, S., Jayamanne, S., Kandasamy, J., Eddleston, M., Buckley, N. A., Dawson, A. H., & Roberts, D. M. (2009). Acute human self-poisoning with imidacloprid compound: a neonicotinoid insecticide. *PloS one*, 4(4), e5127. https://doi.org/10.1371/journal.pone.0005127

Mundhe, S. A., Birajdar, S. V., Chavan, S. S., & Pawar, N. R. (2017). Imidacloprid Poisoning: An Emerging Cause of Potentially Fatal Poisoning. *Indian Journal of Critical Care Medicine : Peer-Reviewed, Official Publication of Indian Society of Critical Care Medicine,* 21(11), 786–788. https://doi.org/10.4103/ijccm.IJCCM_152_17

Muzembo, B. A., Iwai-Shimada, M., Isobe, T., Arisawa, K., Shima, M., Fukushima, T., & Nakayama, S. F. (2019). Dioxins levels in human blood after implementation of measures against dioxin exposure in Japan. *Environmental Health and Preventive Medicine,* 24(1), 6. https://doi.org/10.1186/s12199-018-0755-7

Nassan, F. L., Mínguez-Alarcón, L., Williams, P. L., Dadd, R., Petrozza, J. C., Ford, J. B., Calafat, A. M., Hauser, R., & EARTH Study Team (2019). Urinary triclosan concentrations and semen quality among men from a fertility clinic. *Environmental Research,* 177, 108633. https://doi.org/10.1016/j.envres.2019.108633

National Academy of Sciences. (2008). 2 Phthalate Exposure Assessment in Humans. In: Phthalates and Cumulative Risk Assessment: The Tasks Ahead. https://www.ncbi.nlm.nih.gov/books/NBK215044/

National Institute of Environmental Health Science. (2021). *Bisphenol A (BPA).* https://www.niehs.nih.gov/health/topics/agents/sya-bpa/index.cfm

National Research Council (US) Committee on Measuring Lead in Critical Populations. Measuring Lead Exposure in Infants, Children, and Other Sensitive Populations. Washington (DC): National Academies Press (US). (1993). 2, Adverse Health Effects of Exposure to Lead. Available from: https://www.ncbi.nlm.nih.gov/books/NBK236465

New Jersey Departament of Health. (2010). *Hazardous Substance Fact Sheet*. https://nj.gov/health/eoh/rtkweb/documents/fs/0709.pdf

Oregon State University. (2010). *Imidacloprid*. http://npic.orst.edu/factsheets/imidagen.html#exposed

Papoutsopoulou, S., Satsangi, J., Campbell, B. J., & Probert, C. S.. (2020). Review article: impact of cigarette smoking on intestinal inflammation-direct and indirect mechanisms. *Alimentary Pharmacology & Therapeutics*, 51(12), 1268–1285. https://doi.org/10.1111/apt.15774

Pan, X., Hu, J., Xia, W., Zhang, B., Liu, W., Zhang, C., Yang, J., Hu, C., Zhou, A., Chen, Z., Cao, J., Zhang, Y., Wang, Y., Huang, Z., Lv, B., Song, R., Zhang, J., Xu, S., & Li, Y. (2017). Prenatal chromium exposure and risk of preterm birth: a cohort study in Hubei, China. *Scientific Reports*, 7(1), 3048. https://doi.org/10.1038/s41598-017-03106-z

Pan American Health Organization. (w.d.). *Arsenic*. https://www3.paho.org/hq/index.php?option=com_content&view=article&id=9201:2013-arsenic&Itemid=40132&lang=es

Parvez, S., Gerona, R. R., Proctor, C., Friesen, M., Ashby, J. L., Reiter, J. L., Lui, Z., & Winchester, P. D. (2018). Glyphosate exposure in pregnancy and shortened gestational length: a prospective Indiana birth cohort study. *Environmental Health : a Global Access Science Source*, 17(1), 23. https://doi.org/10.1186/s12940-018-0367-0

Perkins, J. T., Petriello, M. C., Newsome, B. J., & Hennig, B.. (2016). Polychlorinated biphenyls and links to cardiovascular disease. *Environmental Science and Pollution Research*, 23(3), 2160–2172. https://doi.org/10.1007/s11356-015-4479-6

Persson, E. C., Graubard, B. I., Evans, A. A., London, W. T., Weber, J. P., LeBlanc, A., Chen, G., Lin, W., & McGlynn, K. A. (2012). Dichlorodiphenyltrichloroethane and risk of hepatocellular carcinoma. *International Journal of Cancer*, 131(9), 2078–2084. https://doi.org/10.1002/ijc.27459

Pesatori, A. C., Consonni, D., Rubagotti, M., Grillo, P., & Bertazzi, P. A. (2009). Cancer incidence in the population exposed to dioxin after the "Seveso accident": twenty years of follow-up. *Environmental health: a global access science source*, 8, 39. https://doi.org/10.1186/1476-069X-8-39

Poothong, S., Papadopoulou, E., Padilla-Sánchez, J. A., Thomsen, C., & Haug, L. S. (2020). Multiple pathways of human exposure to poly- and perfluoroalkyl substances (PFASs): From external exposure to human blood. *Environment International*, 134, 105244. https://doi.org/10.1016/j.envint.2019.105244

Pozuelos, G. L., Kagda, M. S., Schick, S., Girke, T., Volz, D. C., & Talbot, P. (2019). Experimental Acute Exposure to Thirdhand Smoke and Changes in the Human Nasal Epithelial Transcriptome: A Randomized Clinical Trial. *JAMA network open*, 2(6), e196362. https://doi.org/10.1001/jamanetworkopen.2019.6362

Rafati Rahimzadeh, M., Rafati Rahimzadeh, M., Kazemi, S., & Moghadamnia, A. A. (2017). Cadmium toxicity and treatment: An update. *Caspian Journal of Internal Medicine*, 8(3), 135–145. https://doi.org/10.22088/cjim.8.3.135

Rezg, R., El-Fazaa, S., Gharbi, N., & Mornagui, B. (2014). Bisphenol A and human chronic diseases: current evidences, possible mechanisms, and future perspectives. *Environment Internationa*l, 64, 83–90. https://doi.org/10.1016/j.envint.2013.12.007

Rivers, A. R., Burns, A. S., Chan, L. K., & Moran, M. A. (2016). Experimental Identification of Small Non-Coding RNAs in the Model Marine Bacterium Ruegeria pomeroyi DSS-3. *Frontiers in Microbiology*, 7, 380. https://doi.org/10.3389/fmicb.2016.00380

Schecter, A., Stanley, J., Boggess, K., Masuda, Y., Mes, J., Wolff, M., Fürst, P., Fürst, C., Wilson-Yang, K., & Chisholm, B. (1994). Polychlorinated biphenyl levels in the tissues of exposed and nonexposed humans. *Environmental Health Perspectives,* 102 Suppl 1(Suppl 1), 149–158. https://doi.org/10.1289/ehp.94102s1149

Shankar, A., Xiao, J., & Ducatman, A.. (2012). Perfluorooctanoic Acid and Cardiovascular Disease in US Adults. *Archives of Internal Medicine*, 172(18), 1397. https://doi.org/10.1001/archinternmed.2012.3393

Shanker, A.D. & Venkateswarlu, B. (2011). Chromium: Environmental Pollution, Health Effects and Mode of Action. *Encyclopedia of Environmental Health.* 650-659, https://doi.org/10.1016/B978-0-444-52272-6.00390-1

Sharma, M. K., & Kumar, M.. (2020). Sulphate contamination in groundwater and its remediation: an overview. *Environmental Monitoring and Assessment*, 192(2). https://doi.org/10.1007/s10661-019-8051-6

Shelnutt, S., Kind, J., & Allaben, W. (2013). Bisphenol A: Update on newly developed data and how they address NTP's 2008 finding of "Some Concern". *Food and chemical toxicology : an international journal published for the British Industrial Biological Research Association*, 57, 284–295. https://doi.org/10.1016/j.fct.2013.03.027

Shvedova, A. A., Yanamala, N., Kisin, E. R., Khailullin, T. O., Birch, M. E., & Fatkhutdinova, L. M.. (2016). Integrated Analysis of Dysregulated ncRNA and mRNA Expression Profiles in Humans Exposed to Carbon Nanotubes. *PLOS ONE*, 11(3), e0150628. https://doi.org/10.1371/journal.pone.0150628

Skarha, J., Mínguez-Alarcón, L., Williams, P. L., Korevaar, T., de Poortere, R. A., Broeren, M., Ford, J. B., Eliot, M., Hauser, R., & Braun, J. M. (2019). Cross-sectional associations between urinary triclosan and serum thyroid function biomarker concentrations in women. *Environment International*, 122, 256–262. https://doi.org/10.1016/j.envint.2018.11.015

Steenland, K., & Boffetta, P. (2000). Lead and cancer in humans: where are we now?. *American Journal of Industrial Medicine*, 38(3), 295–299. https://doi.org/10.1002/1097-0274(200009)38:3<295::aid-ajim8>3.0.co;2-l

Steenland, K., Fletcher, T., Stein, C. R., Bartell, S. M., Darrow, L., Lopez-Espinosa, M. J., Barry Ryan, P., & Savitz, D. A. (2020). Review: Evolution of evidence on PFOA and health following the assessments of the C8 Science Panel. *Environment International,* 145, 106125. https://doi.org/10.1016/j.envint.2020.106125

Sun, D., Zhao, T., Long, K., Wu, M., & Zhang, Z. (2021). Triclosan down-regulates fatty acid synthase through microRNAs in HepG2 cells. *European Journal of Pharmacology*, 907, 174261. https://doi.org/10.1016/j.ejphar.2021.174261

Sun, L., Xu, A., Li, M., Xia, X., Li, P., Han, R., Fei, G., Zhou, S., & Wang, R. (2021). Effect of Methylation Status of lncRNA-MALAT1 and MicroRNA-146a on Pulmonary Function and Expression Level of COX2 in Patients With Chronic Obstructive Pulmonary Disease. *Frontiers in Cell and Developmental Biology*, 9, 667624. https://doi.org/10.3389/fcell.2021.667624

Tang, F., Wang, H., Chen, E., Bian, E., Xu, Y., Ji, X., Yang, Z., Hua, X., Zhang, Y., & Zhao, B. (2019). LncRNA-ATB promotes TGF-β-induced glioma cells invasion through NF-κB and P38/MAPK pathway. *Journal of Cellular Physiology*, 234(12), 23302–23314. https://doi.org/10.1002/jcp.28898

Tani, H., Numajiri, A., Aoki, M., Umemura, T., & Nakazato, T.. (2019). Short-lived long noncoding RNAs as surrogate indicators for chemical stress in HepG2 cells and their degradation by nuclear RNases. *Scientific Reports*, 9(1). https://doi.org/10.1038/s41598-019-56869-y

University of Tennessee. (2020). Toxicity Profiles. Formal Toxicity Summary for Cadmium. https://rais.ornl.gov/tox/profiles/cadmium.html

University of Tennessee. (2020a). Toxicity Profiles. Formal Toxicity Summary for Chromium. https://rais.ornl.gov/tox/profiles/chromium.html

United States Environmental Protection Agency (EPA). (2021). *Polychlorinated Biphenyls (PCBs)*. https://www.epa.gov/pcbs/learn-about-polychlorinated-biphenyls-pcbs

United States Environmental Protection Agency (EPA). (2021a). *PFOA, PFOS and Other PFAS.* https://www.epa.gov/pfas/pfas-explained

US Department of Health and Human Services. (2020). Toxicological Profile for Glyphosate. Agency for toxic Subtances and Disease Registry. https://www.atsdr.cdc.gov/toxprofiles/tp214.pdf

Wahlang, B., Petriello, M. C., Perkins, J. T., Shen, S., & Hennig, B.. (2016). Polychlorinated biphenyl exposure alters the expression profile of microRNAs associated with vascular diseases. *Toxicology in Vitro*, 35, 180–187. https://doi.org/10.1016/j.tiv.2016.06.001

Wang, W., Shim, Y. K., Michalek, J. E., Barber, E., Saleh, L. M., Choi, B. Y., Wang, C. P., Ketchum, N., Costello, R., Marti, G. E., Vogt, R. F., Landgren, O., & Calvo, K. R. (2020). Serum microRNA profiles among dioxin exposed veterans with monoclonal gammopathy of undetermined significance. *Journal of Toxicology and Environmental Health. Part A*, 83(7), 269–278. https://doi.org/10.1080/15287394.2020.1749919

Wani, A. L., Ara, A., & Usmani, J. A. (2015). Lead toxicity: a review. *Interdisciplinary Toxicology*, 8(2), 55–64.

https://doi.org/10.1515/intox-2015-0009

Weatherly, L. M., & Gosse, J. A. (2017). Triclosan exposure, transformation, and human health effects. Journal of toxicology and environmental health. Part B, *Critical Reviews*, 20(8), 447–469. https://doi.org/10.1080/10937404.2017.1399306

Woeller, C. F., Thatcher, T. H., Thakar, J., Cornwell, A., Smith, M. R., Jones, D. P., Hopke, P. K., Sime, P. J., Krahl, P., Mallon, T. M., Phipps, R. P., & Utell, M. J. (2019). Exposure to Heptachlorodibenzo-p-dioxin (HpCDD) Regulates microRNA Expression in Human Lung Fibroblasts. *Journal of Occupational and Environmental Medicine*, 61 Suppl 12(Suppl 12), S82–S89. https://doi.org/10.1097/JOM.0000000000001691

World Health Organization. Regional Office for Europe, Copenhagen, Denmark. (2000). *Chapter 6.4 Chromium.* https://www.euro.who.int/__data/assets/pdf_file/0017/123074/AQG2ndEd_6_4Chromium.PDF

World Health Organization. (2010). Preventing disease through healthy environments exposure to dioxins and dioxin-like substances: a major public health concern. https://www.who.int/ipcs/features/dioxins.pdf

World Health Organization. (2019). Preventing disease through healthy environments. Exposure to cadmium: a major public health concern. https://apps.who.int/iris/bitstream/handle/10665/329480/WHO-CED-PHE-EPE-19.4.3-eng.pdf?ua=1

World Health Organization. (2022). IARC Monograph on Glyphosate. https://www.iarc.who.int/featured-news/media-centre-iarc-news-glyphosate/

World Health Organization, International Agency for Research on Cancer. (2022). *Triclosan (TCS) (Compound).* http://exposome-explorer.iarc.fr/compounds/1420

World Health Organization. Regional Office for Europe, Copenhagen, Denmark. (2000a). Chapter 5.10 Polychlorinated biphenyls (PCBs). https://www.euro.who.int/__data/assets/pdf_file/0016/123064/AQG2ndEd_5_10PCBs.PDF

World Health Organization. Regional Office for Europe, Copenhagen, Denmark. (2000). Chapter 8.1 Environmental tobacco smoke. https://www.euro.who.int/__data/assets/pdf_file/0003/123087/AQG2ndEd_8_1ETS.PDF

Xu, M., Yu, Z., Hu, F., Zhang, H., Zhong, L., Han, L., An, Y., Zhu, B., & Zhang, H. (2017). Identification of differential plasma miRNA profiles in Chinese workers with occupational lead exposure. *Bioscience Reports*, 37(5), BSR20171111. https://doi.org/10.1042/BSR20171111

Xu, Y., Jurkovic-Mlakar, S., Li, Y., Wahlberg, K., Scott, K., Pineda, D., Lindh, C. H., Jakobsson, K., & Engström, K. (2020). Association between serum concentrations of perfluoroalkyl substances (PFAS) and expression of serum microRNAs in a cohort highly exposed to PFAS from drinking water. *Environment International*, 136, 105446. https://doi.org/10.1016/j.envint.2019.105446

Yan, W., Yue, H., Ji, X., Li, G., & Sang, N. (2020). Prenatal NO2 exposure and neurodevelopmental disorders in offspring mice: Transcriptomics reveals sex-dependent changes in cerebral gene expression. *Environment International*, 138, 105659. https://doi.org/10.1016/j.envint.2020.105659

Zefferino, R., Piccoli, C., Ricciardi, N., Scrima, R., & Capitanio, N. (2017). Possible Mechanisms of Mercury Toxicity and Cancer Promotion: Involvement of Gap Junction Intercellular Communications and Inflammatory Cytokines. *Oxidative Medicine and Cellular Longevity*, 2017, 7028583. https://doi.org/10.1155/2017/7028583

Zhang, B. Z., Hu, G. L., Lu, L. Y., Hu, S. F., Li, Y. S., Su, X., Dong, W. Y., Zhen, C. A., Liu, R. Q., Kong, F. B., Shi, M. W., & Chen, X. L. (2021). Identification of differentially expressed microRNAs under imidacloprid exposure in Sitobion miscanthi. *Pesticide Biochemistry and Physiology*, 177, 104885. https://doi.org/10.1016/j.pestbp.2021.104885

Zhou, Y; He, L; Liu, X.D; Guan, H; Li, Y; Huang, R.X. & Zhou, P.K. (2019). Integrated Analysis of lncRNA and mRNA Transcriptomes Reveals New Regulators of Ubiquitination and the Immune Response in Silica-Induced Pulmonary Fibrosis. *Biomed Research International,* 2019, https://doi.org/10.1155/2019/6305065.

Zota, A. R., Geller, R. J., Vannoy, B. N., Marfori, C. Q., Tabbara, S., Hu, L. Y., Baccarelli, A. A., & Moawad, G. N.. (2020). Phthalate Exposures and MicroRNA Expression in Uterine Fibroids: The FORGE Study. *Epigenetics Insights*, 13, 251686572090405. https://doi.org/10.1177/2516865720904057
